# Clinician preferences and decision-making patterns in the rehabilitation of severe maxillary atrophy: an international cross-sectional survey using a standardized cawood and howell class VI case

**DOI:** 10.3389/froh.2026.1903798

**Published:** 2026-08-04

**Authors:** Giulio Gasparini, Mattia Todaro, Edoardo Papa, Marco Gaetano Amato, Paolo De Angelis, Alessandro Moro, Carlo Lajolo, Gianluca Nicolai, Edoardo Rella, Roberto Boniello, Xavier Moix Gil, Edoardo Staderini, Sofia Gasparini, Federico Perquoti, Gianmarco Spinozzi, Veronica Santoro, Daniele Di Carlo, Giuliano Ascani, Paolo Francesco Manicone, Maurizio Diego Foresti, Maurizio Perugini, Francesca Azzuni, The collaborative Working Group, Martinna Bertolini, Gianmarco Saponaro

**Affiliations:** 1 Pre-prosthetic Surgery Unit, Fondazione Policlinico Agostino Gemelli IRCCS Hospital, Rome, Italy; 2 Maxillo-Facial Surgery Unit, IRCSS “A. Gemelli” Foundation-Catholic University of the Sacred Heart, Rome, Italy; 3 Department of Emergency and Acceptance, UOSD Maxillofacial Surgery, University of Rome Tor Vergata, Rome, Italy; 4 Department of Head and Neck, Oral Surgery and Implantology Unit, Institute of Clinical Dentistry, Catholic University of the Sacred Heart, Polyclinic Foundation, Rome, Italy; 5 Head and Neck Department, “Fondazione Policlinico Universitario A. Gemelli–IRCCS,” School of Dentistry, Università Cattolica del Sacro Cuore, Rome, Italy; 6 Department of Head and Neck and Sensory Organs, Division of Oral Surgery and Implantology, Fondazione Policlinico Universitario A. Gemelli IRCCS-Università Cattolica del Sacro Cuore, Rome, Italy; 7 IRCCS, Fondazione Policlinico Universitario Agostino Gemelli, Roma, Italy; 8 Department of Orthodontics, Università Cattolica del Sacro Cuore, Rome, Italy; 9 Division of Maxillo-Facial Surgery, Department of Medicine, Surgery and Dentistry, ASST Santi Paolo e Carlo Hospital, University of Milan, Milan, Italy; 10 Department of Maxillofacial Surgery, Spirito Santo Hospital, Pescara, Italy; 11 Division of Prosthodontics - Institute of Clinical Dentistry, Department of Neuroscience, Sensory Organs and Chest, Fondazione Policlinico Universitario A. Gemelli – IRCCS, School of Dentistry, Universita’ Cattolica del Sacro Cuore., Roma, Italy; 12 Maxillo-Facial Surgery Unit, Ospedale SS. Trinità, Cagliari, Italy; 13 Department of Maxillo Facial Surgery, Santa Rosa Hospital Viterbo, Viterbo, Italy

**Keywords:** bone grafting, Cawood and Howell Class VI, clinician preferences, patient-specific implants (PSI), pterygoid implants, severe maxillary atrophy, treatment decision—making, zygomatic implants

## Abstract

**Background:**

Severe maxillary atrophy represents a complex clinical condition requiring individualized implant-prosthetic rehabilitation strategies. Although several therapeutic approaches are available, including graftless implant solutions, regenerative procedures, and hybrid strategies, the decision-making rationale underlying clinicians' preferences remains incompletely defined.

**Objective:**

This international cross-sectional survey aimed to investigate clinician-reported decision-making profiles, perceived advantages, and expected outcomes associated with different therapeutic strategies for the rehabilitation of severe maxillary atrophy.

**Materials and methods:**

An international questionnaire-based survey was conducted among clinicians with experience in complex implant-prosthetic rehabilitation. A total of 178 questionnaires were submitted. After exclusion of respondents reporting fewer than five complex rehabilitations per year, 155 eligible responses were included in the final analysis. Participants evaluated a standardized anonymized CBCT clinical case of severe maxillary atrophy classified as Cawood and Howell Class VI and selected their preferred therapeutic strategy. According to this choice, respondents were allocated into three groups: alternative implant strategies, regenerative approach with bone grafting, and hybrid/combined strategy. Descriptive statistics were used to summarize clinician-reported perceptions regarding safety, predictability, immediate loading, expected implant survival, treatment duration, aesthetic and functional outcomes, maintainability, morbidity, maintenance costs, patient satisfaction, and quality-of-life improvement.

**Results:**

Alternative implant strategies were the most frequently selected approach, reported by 83 clinicians (53.5%), followed by regenerative approaches with bone grafting in 45 cases (29.0%) and hybrid/combined strategies in 27 cases (17.4%). Alternative strategies were more frequently associated with immediate loading and shorter treatment duration, whereas regenerative approaches were more commonly perceived as advantageous for aesthetic volume reconstruction and soft-tissue support. Expected five-year implant survival, masticatory function, maintenance burden, and clinician-reported patient satisfaction were generally perceived as favorable across all groups.

**Conclusions:**

Within the limitations of a perception-based survey design and a relatively limited sample size, the findings suggest that therapeutic decision-making in severe maxillary atrophy is not based on a rigid hierarchy of techniques. Instead, clinicians appear to balance treatment timing, surgical morbidity, anatomical reconstruction, prosthetic objectives, aesthetic expectations, maintainability, and patient-related priorities. These results should not be interpreted as evidence of clinical superiority of one approach over another. Further prospective multicenter studies using standardized clinical endpoints and validated patient-reported outcome measures are needed to clarify the comparative effectiveness of these strategies.

## 1 Introduction

The rehabilitation of patients affected by severe maxillary atrophy remains one of the most challenging conditions in contemporary oral and maxillofacial surgery (1). In patients classified as Cawood and Howell Class VI (2), the severe reduction of residual bone volume, combined with maxillary sinus pneumatization, nasal floor expansion, and soft tissue limitations, frequently prevents the use of conventional implant-supported rehabilitation strategies. In these cases, treatment planning must balance functional restoration, prosthetic stability, aesthetic rehabilitation, surgical invasiveness, and long-term predictability.

Traditionally, rehabilitation of severe maxillary atrophy has relied on regenerative procedures involving autologous or heterologous bone grafts (3–5), including onlay grafts, interpositional grafts, and microvascular reconstruction in the most advanced cases (6, 7). These techniques may allow restoration of bone volume suitable for conventional implant placement; however, they are frequently associated with increased morbidity, prolonged treatment time, donor site complications, higher costs, and variable graft resorption (8–10).

Over the last two decades, the development of graftless approaches has significantly modified the therapeutic landscape. Zygomatic (11) and pterygoid (12) implants, together with digitally designed CAD/CAM-customized subperiosteal implants (13–15), have progressively emerged as alternative treatment options for patients with limited residual bone availability. These techniques aim to reduce surgical invasiveness and rehabilitation time while enabling immediate or early prosthetic loading. Simultaneously, advances in digital planning, guided surgery, and additive manufacturing technologies have contributed to expanding personalized treatment strategies in severe maxillary atrophy rehabilitation.

Despite these developments, considerable heterogeneity still exists regarding treatment selection, surgical philosophy, and prosthetic planning (16, 17). Current literature remains fragmented, frequently based on single-center experiences, retrospective analyses, or technique-specific case series. Furthermore, limited information is available regarding how experienced clinicians currently approach therapeutic decision-making in severe maxillary atrophy, particularly when multiple valid surgical options coexist. In the absence of definitive comparative evidence demonstrating the superiority of a single therapeutic strategy, treatment selection often relies on clinician experience, anatomical considerations, prosthetic objectives, and patient-specific factors.

Understanding current international trends and expert preferences may provide useful insight into contemporary clinical reasoning, perceived advantages and limitations of different approaches, and evolving treatment paradigms. In this context, expert-based surveys may represent a valuable tool to explore how clinicians integrate anatomical, functional, prosthetic, and patient-related factors into their therapeutic strategies. To our knowledge, few international studies have specifically explored how experienced clinicians approach therapeutic decision-making when presented with the same standardized severe maxillary atrophy case.

Therefore, the aim of the present international cross-sectional survey was to investigate current trends, treatment preferences, clinician-reported perceptions, and decision-making criteria adopted by experienced clinicians in the rehabilitation of severe maxillary atrophy through the evaluation of a standardized anonymized CBCT clinical case2.

## 2 Materials and methods

### 2.1 Study design and general objectives

The present study was designed as an international cross-sectional survey aimed at investigating therapeutic preferences in the implant-prosthetic rehabilitation of severe maxillary atrophy, classified as Cawood and Howell Class VI.

The study was based on a structured online questionnaire developed to collect clinician-reported data regarding treatment preferences, surgical and prosthetic planning strategies, and decision-making criteria adopted by clinicians experienced in the management of severe maxillary atrophy.

The primary objective was to describe the distribution of preferred rehabilitation strategies among experienced clinicians when presented with the same standardized anonymized CBCT case of severe maxillary atrophy.

Secondary objectives included the evaluation of factors influencing therapeutic choice and clinician-reported perceptions regarding different rehabilitation strategies, including regenerative procedures, alternative implant approaches, hybrid treatment concepts, expected implant survival, aesthetic outcomes, treatment duration, postoperative discomfort, maintenance burden, and patient satisfaction.

The study aimed to collect expert opinions and clinician-reported perceptions rather than direct patient outcome data.

### 2.2 Participants and eligibility criteria

The study population consisted of oral surgeons, maxillofacial surgeons, implantologists, prosthodontists, and clinicians involved in advanced implant-prosthetic rehabilitation.

Participants were recruited through direct invitations, scientific mailing lists, professional societies, and international academic and professional networks.

The initial dataset included 178 submitted questionnaires. The predefined eligibility criterion was self-reported experience in complex implant-prosthetic rehabilitation, defined as the treatment of at least five complex rehabilitations per year.

After screening, 23 questionnaires were excluded because respondents reported fewer than five complex rehabilitations per year. The final analysis therefore included 155 eligible responses.

#### 2.2.1 Geographic distribution of participants

Russia, Denmark, Spain, Iran, Italy, Austria, France, Germany, Brazil, Belgium, Chile, Turkey, Albania, India, Australia, Norway, Netherlands, Hungary, Switzerland, Egypt, Libya, USA, Moldova, Sweden, China, Lithuania, Montenegro, Poland, Portugal, Romania, South Africa, South Korea, Uruguay. Europe accounted for the largest proportion of respondents, followed by North America, South America and Asia, while a smaller but significant fraction originated from Africa and Oceania. Regarding professional profile, the majority of participants were maxillofacial surgeons, oral surgeons, or implantologists, with a smaller but notable representation of prosthodontists and dentists with advanced implant rehabilitation expertise.

The distribution by area of activity indicated a predominance of practitioners working in hospital-university centers and specialized private clinics, with a smaller representation from research institutions or postgraduate academic settings.

The final sample was distributed into three therapeutic preference groups according to the technique selected for the standardized clinical case:

- Alternative implant strategies: 83 participants (53.5%)
- Regenerative approaches with bone grafting: 45 participants (29.0%)
- Hybrid/combined strategies: 27 participants (17.4%)

Participation was voluntary, unpaid, and independent from commercial sponsorship.

### 2.3 Questionnaire structure and standardized clinical case

The questionnaire was developed in English using the Google Forms platform to facilitate international dissemination and standardized access.

**Figure 1:**
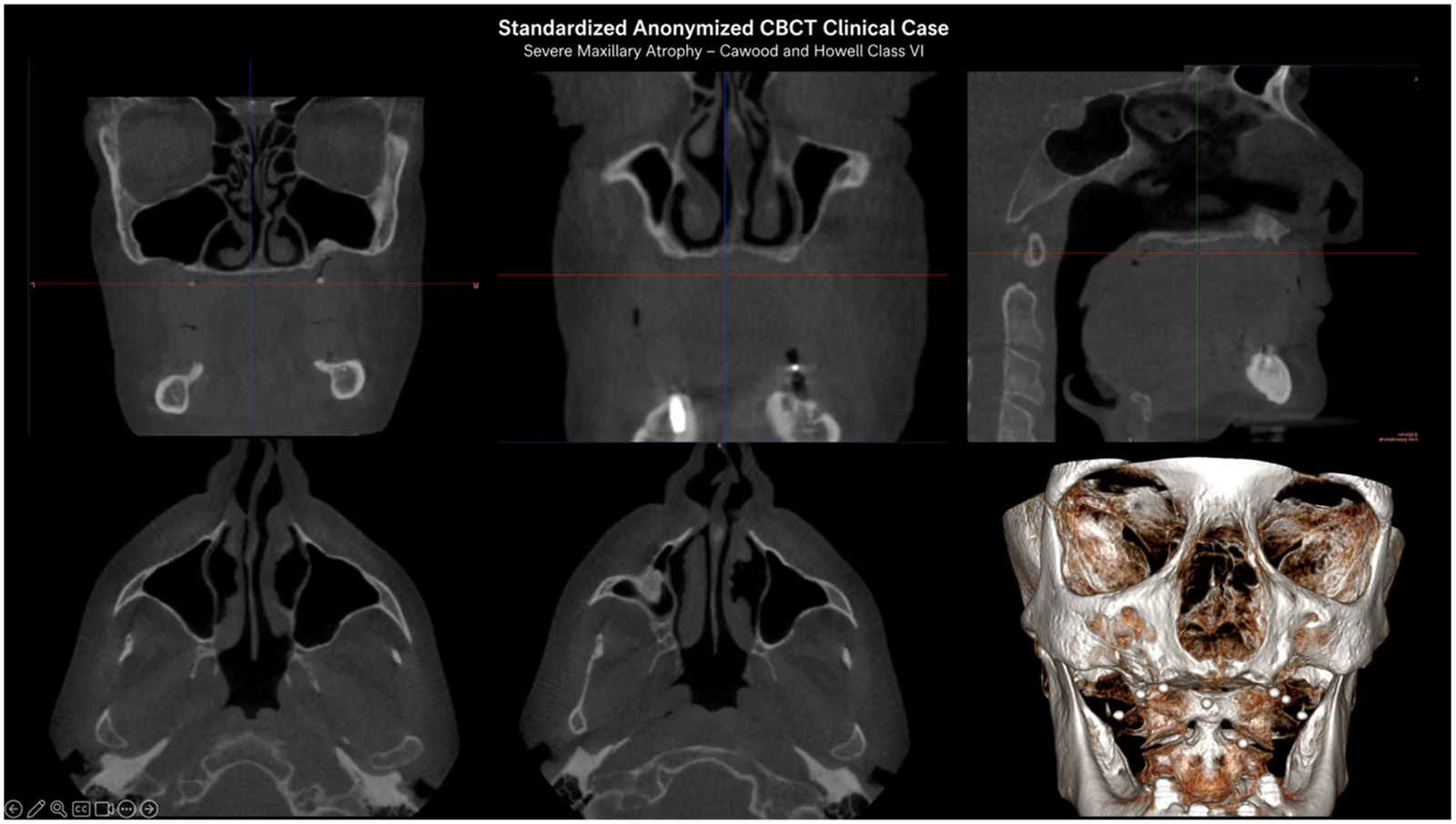
Standardized anonymized CBCT case used for treatment-planning assessment. Representative cone-beam computed tomography (CBCT) images of the standardized clinical case presented to all survey participants. The figure includes coronal sections at different anteroposterior levels, axial sections at different craniocaudal levels, a sagittal reconstruction, and a three-dimensional volume rendering. The images demonstrate severe maxillary atrophy consistent with Cawood and Howell Class VI and served as the radiological basis for therapeutic decision-making within the survey. All patient-identifiable information was removed prior to dissemination.

Participants were presented with a standardized clinical case of severe maxillary atrophy classified as Cawood and Howell Class VI. The case was designed to simulate a complex but realistic implant-prosthetic rehabilitation scenario. Representative images of the anonymized CBCT case are provided in Figure 1.

The questionnaire was structured into three main sections.

The first section collected demographic and professional information, including professional qualification, specialty, workplace setting, geographic area of practice, years of clinical experience, and number of complex rehabilitations performed per year.

The second section presented the standardized clinical case and asked participants to select their preferred therapeutic approach among the following options: alternative implant strategies, including zygomatic, pterygoid, transnasal, tilted, subperiosteal, or patient-specific implants; regenerative approach with bone grafting followed by conventional implantology; or hybrid/combined strategy.

The third section explored therapeutic planning and clinician-reported perceptions regarding the selected approach. The questionnaire used an internal branching structure: after selecting the preferred therapeutic strategy, each participant accessed a set of questions specifically related to the chosen approach. This structure allowed each therapeutic group to be analyzed separately according to the selected therapeutic strategy.

The investigated domains included perceived safety, predictability, tolerability, possibility of immediate loading, expected implant survival at five years, treatment duration, perceived aesthetic outcomes, masticatory function, oral hygiene and maintainability, postoperative discomfort, dropout due to morbidity, annual maintenance costs, clinician-reported patient satisfaction, and perceived quality-of-life improvement.

All questions were formulated as closed-ended items with predefined multiple-choice responses to ensure response standardization and comparability. The full survey questionnaire, including the branching structure according to the selected therapeutic strategy, is provided as Supplementary Material 1.

### 2.4 Questionnaire review and validation

Before international dissemination, the questionnaire underwent internal qualitative review by experienced clinicians involved in maxillofacial surgery, oral surgery, implantology, and prosthetic rehabilitation.

The review process aimed to assess clinical relevance, semantic clarity, logical coherence, and completeness of the investigated domains. Minor wording and structural refinements were introduced before final dissemination.

Formal psychometric validation, including construct validity assessment, factor analysis, or test–retest reliability analysis, was not performed. This was considered appropriate because the questionnaire was designed to describe explicit clinical preferences and decision-making orientations within a standardized clinical scenario, rather than to measure latent psychological constructs.

### 2.5 Data collection procedure

Data were collected through an anonymous online questionnaire hosted on Google Forms and accessible through a direct electronic link.

The survey remained open between July 2025 and November 2025. During this period, the questionnaire was distributed to approximately **500 clinicians** with experience in implant-prosthetic rehabilitation through direct invitations, professional and academic networks, scientific mailing lists, and international professional societies. Responses submitted within the survey period were collected and exported in Microsoft Excel format before being screened for eligibility, completeness, and consistency.

**Figure 2:**
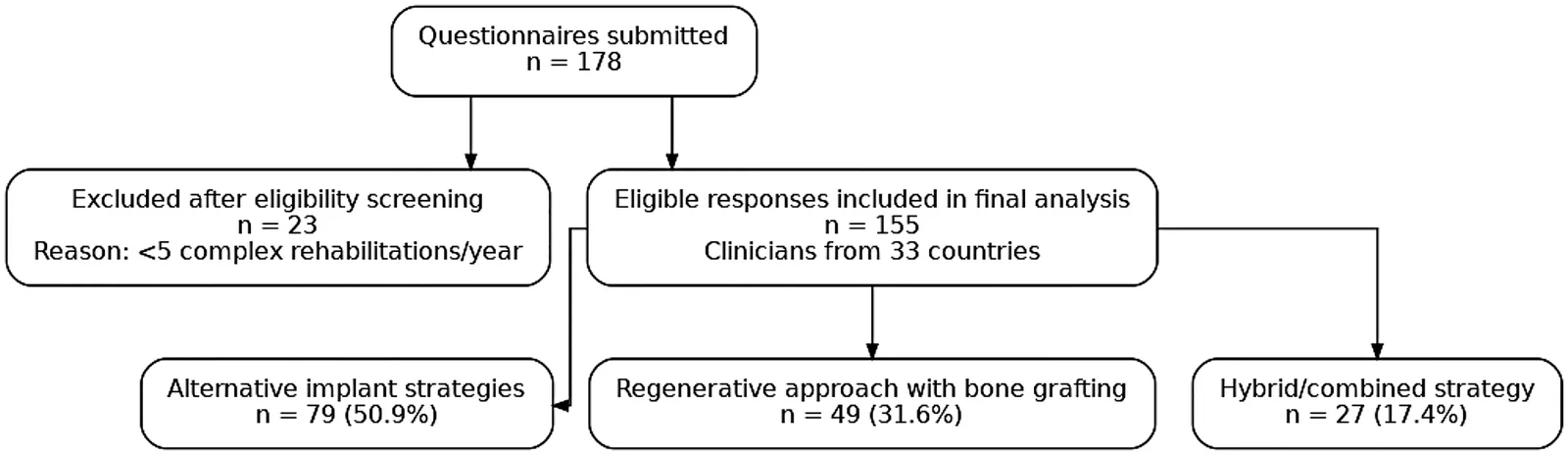
Flowchart of participant selection and therapeutic preference allocation. A total of 178 questionnaires were submitted. After application of the predefined eligibility criterion requiring self-reported experience in at least five complex implant-prosthetic rehabilitations per year, 23 questionnaires were excluded. The final analysis included 155 eligible clinicians from 33 countries. Participants were allocated into three therapeutic preference groups according to the treatment strategy selected for the standardized CBCT case: alternative implant strategies (*n* = 83, 53.5%), regenerative approaches with bone grafting (*n* = 45, 29.0%), and hybrid/combined strategies (*n* = 27, 17.4%).

A total of **178 questionnaires** were received during the study period, corresponding to an overall response rate of **35.6%**. After exclusion of 23 respondents who did not meet the predefined self-reported experience threshold of at least five complex rehabilitations per year, **155 eligible responses** from **33 countries** were included in the final analysis, corresponding to an eligible response rate of **31.0%** (Figure 2).

### 2.6 Outcomes

The primary outcome of the study was the distribution of preferred rehabilitation strategies for the standardized case of severe maxillary atrophy among experienced clinicians.

Secondary outcomes included clinician-reported perceptions and **expectations** regarding the safety, predictability, and tolerability of the selected therapeutic approach; the feasibility of immediate loading with a fixed prosthesis; expected five-year implant survival; overall treatment duration; expected aesthetic outcomes, including smile satisfaction; expected masticatory function; oral hygiene and maintainability; postoperative discomfort; treatment dropout attributable to surgical morbidity; annual maintenance costs; clinician-reported patient satisfaction; and perceived quality-of-life improvement.

All outcomes represent clinicians' expectations and professional judgments based on the evaluation of a standardized clinical case and their own clinical experience. They do not represent measured clinical outcomes, prospective patient data, patient-reported outcome measures (PROMs), validated quality-of-life assessments, or direct comparisons between treatment modalities. Furthermore, because each respondent evaluated only the therapeutic strategy they personally preferred, differences among groups reflect differences in clinician perceptions and expectations rather than objective differences between rehabilitation techniques.

Accordingly, the findings should be interpreted as reflecting contemporary expert opinions, therapeutic preferences, and decision-making patterns, rather than evidence of clinical effectiveness or superiority of any specific rehabilitation strategy.

### 2.7 Statistical analysis

Statistical analysis was descriptive.

Categorical variables were summarized using absolute frequencies and percentages.

Percentages were calculated based on valid responses for each survey item. Because the questionnaire included a branching structure, denominators may vary across survey items and are reported where appropriate.

No inferential statistical tests or multivariate models were applied, as the aim of the present analysis was descriptive and exploratory, focusing on clinician-reported preferences and perceptions rather than hypothesis testing.

Given the exploratory, perception-based design of the survey and the branching structure of the questionnaire, inferential statistical comparisons between therapeutic preference groups were intentionally not performed. Since each respondent evaluated only the therapeutic strategy they personally selected as their preferred approach for the standardized clinical scenario, the groups did not represent independent evaluations of competing treatment modalities. Accordingly, the analysis was limited to descriptive statistics, which were considered the most appropriate for addressing the study objectives.

Data management and descriptive analyses were performed using Microsoft Excel.

### 2.8 Ethical considerations and data management

Responses were collected anonymously and analyzed in aggregated form.

No patient-identifiable data, human images, or direct patient-reported outcomes were collected. The standardized clinical case used in the survey was anonymized before dissemination.

Participation was voluntary, and completion of the questionnaire was considered as implied consent to participate.

Because the study involved an anonymous survey of healthcare professionals, did not collect identifiable personal data, and did not involve patient recruitment or clinical interventions, formal Institutional Review Board or Ethics Committee approval was not considered necessary according to applicable institutional and international recommendations.

The study was conducted in accordance with the principles of the Declaration of Helsinki.

Data were exported in Microsoft Excel format and stored on password-protected devices in compliance with applicable data protection regulations.

### 2.9 Authorship and collaborative group

Participation in the survey or contribution to survey dissemination did not automatically imply authorship.

Authorship was assigned in accordance with the recommendations of the International Committee of Medical Journal Editors (ICMJE) and was limited to contributors who fulfilled the authorship criteria and agreed to take responsibility for the published work.

Individuals who contributed to survey dissemination, professional networking, or study promotion but did not meet all authorship requirements, or who declined authorship, were acknowledged where appropriate.

### 2.10 Potential sources of bias

Several limitations inherent to international expert-based surveys should be acknowledged.

Selection bias may have occurred because recruitment was performed through academic, scientific, and professional networks, potentially leading to overrepresentation of highly experienced clinicians, university-affiliated professionals, and high-volume referral centers.

Participation was voluntary, introducing the possibility of self-selection bias, with increased participation from clinicians particularly interested in advanced implant rehabilitation.

The predefined eligibility threshold of at least five complex rehabilitations per year strengthened the expert-based nature of the sample but may have further increased the representation of high-volume clinicians.

In addition, the survey reflects clinician-reported perceptions and decision-making preferences rather than direct patient outcomes or prospective clinical data. Therefore, findings should be interpreted as representative of current expert opinions and international therapeutic trends, rather than as evidence of clinical superiority of any specific technique.

Despite these limitations, the inclusion of experienced clinicians from 33 countries and different professional backgrounds provides a broad overview of contemporary decision-making patterns in the rehabilitation of severe maxillary atrophy.

## 3 Results

The following sections report the main questionnaire domains according to the structure of the survey and the therapeutic strategy selected by respondents.

### 3.1 Sample characteristics

A total of 178 questionnaires were submitted. After application of the predefined eligibility criterion requiring self-reported experience in at least five complex implant-prosthetic rehabilitations per year, 23 questionnaires were excluded. The final analysis therefore included 155 eligible clinicians.

Eligible responses were obtained from clinicians practicing in 33 countries, supporting the international nature of the survey.

Based on the preferred therapeutic strategy selected for the standardized anonymized CBCT case, participants were allocated into three therapeutic preference groups:

- Alternative implant strategies: 83 participants (53.5%)
- Regenerative approaches with bone grafting: 45 participants (29.0%)
- Hybrid/combined strategies: 27 participants (17.4%)

The participant selection process and therapeutic group allocation are summarized in Figure 2.

### 3.2 Therapeutic preferences

Alternative implant strategies represented the most frequently selected therapeutic option for the standardized case of severe maxillary atrophy, accounting for more than half of all responses. Regenerative approaches with bone grafting represented the second most frequently selected strategy, while hybrid or combined approaches accounted for a smaller but clinically relevant proportion of responses.

Overall, these findings indicate that many respondents selected graftless and alternative implant-based solutions for the standardized clinical scenario, while regenerative and hybrid approaches were also frequently selected. Nevertheless, regenerative and hybrid approaches continue to play an important role in contemporary treatment planning, reflecting the absence of a single universally preferred therapeutic strategy among experts.

### 3.3 Perceived safety, predictability, and tolerability

Perceptions of safety, predictability, and tolerability differed among the three therapeutic preference groups.

In the alternative implant strategies group, 67 of 83 respondents (80.7%) considered the selected technique more predictable, 50 (60.2%) considered it well tolerated, and 46 (55.4%) considered it safer.

In the regenerative group, 32 of 45 respondents (71.1%) considered the technique more predictable, 21 (46.7%) considered it safer, and 15 (33.3%) considered it well tolerated.

In the hybrid group, 11 of 27 respondents (40.7%) indicated good tolerability, while 8 respondents (29.6%) considered the approach more predictable and 8 (29.6%) considered it safer (Table 1).

**Table 1:** Reasons supporting the preferred treatment strategy among respondents.

Treatment strategy	Respondents, *n*	More predictable	Safer	Well tolerated
Alternative implant strategies	83	67 (80.7)	46 (55.4)	50 (60.2)
Regenerative approaches with bone grafting	45	32 (71.1)	21 (46.7)	15 (33.3)
Hybrid/combined strategies	27	8 (29.6)	8 (29.6)	11 (40.7)

Overall, respondents selecting alternative implant strategies more frequently perceived their preferred approach as predictable and well tolerated. Regenerative approaches were also commonly regarded as predictable, whereas hybrid approaches showed a more heterogeneous distribution of perceptions. These findings reflect clinician-reported opinions and should not be interpreted as comparative evidence of clinical performance.

### 3.4 Possibility of immediate loading

Clear differences emerged regarding the possibility of immediate loading with a fixed prosthesis.

In the alternative implant strategies group, 68 of 83 respondents (81.9%) reported that immediate loading is almost always possible, 13 (15.7%) considered it feasible only in selected cases, and 2 (2.4%) stated that it is not routinely planned.

In the regenerative group, 21 of 45 respondents (46.7%) indicated that immediate loading is not planned, 22 (48.9%) considered it possible only in selected cases, and only 2 (4.4%) reported that it is generally applicable.

In the hybrid group, 15 of 27 respondents (55.6%) indicated that immediate loading is possible only in selected cases, 7 (25.9%) reported that it is generally feasible, and 5 (18.5%) stated that it is not routinely planned (Table 2).

**Table 2:** Feasibility of immediate loading according to preferred treatment strategy.

Treatment strategy	*n*	Immediate loading routinely feasible	Feasible only in selected cases	Not routinely planned
Alternative implant strategies	83	68 (81.9%)	13 (15.7%)	2 (2.4%)
Regenerative approaches with bone grafting	45	2 (4.4%)	22 (48.9%)	21 (46.7%)
Hybrid/combined strategies	27	7 (25.9%)	15 (55.6%)	5 (18.5%)

Among respondents selecting alternative implant strategies, immediate loading was more frequently considered feasible, whereas respondents selecting regenerative approaches more commonly associated their preferred strategy with delayed loading protocols. Hybrid approaches showed an intermediate pattern, with most respondents considering immediate loading feasible only under selected clinical conditions.

### 3.5 Expected five-year implant survival

Expected five-year implant survival was generally favorable across all therapeutic preference groups, although the distribution of responses differed.

In the alternative implant strategies group, 48 of 83 respondents (57.8%) estimated implant survival above 95%, 33 (39.8%) estimated survival between 85% and 94%, and 2 (2.4%) estimated survival below 85%.

In the regenerative group, 18 of 45 respondents (40.0%) estimated survival above 95%, 26 (57.8%) estimated survival between 85% and 94%, and 1 (2.2%) estimated survival below 85%.

In the hybrid group, 5 of 27 respondents (18.5%) estimated survival above 95%, 20 (74.1%) estimated survival between 85% and 94%, and 2 (7.4%) estimated survival below 85% (Table 3).

**Table 3:** Estimated implant survival according to preferred treatment strategy.

Treatment strategy	*n*	>95%	85%–94%	<85%
Alternative implant strategies	83	48 (57.8%)	33 (39.8%)	2 (2.4%)
Regenerative approaches with bone grafting	45	18 (40.0%)	26 (57.8%)	1 (2.2%)
Hybrid/combined strategies	27	5 (18.5%)	20 (74.1%)	2 (7.4%)

Respondents selecting alternative implant strategies more frequently estimated implant survival above 95%, whereas respondents selecting regenerative and hybrid approaches more commonly estimated survival between 85% and 94%. These results reflect clinician-reported expectations and should not be interpreted as prospective clinical survival data.

### 3.6 Overall treatment duration

Perceived treatment duration differed substantially among the three therapeutic preference groups.

In the alternative implant strategies group, 69 of 83 respondents (83.1%) reported functional loading within 72 h, 11 (13.3%) reported a treatment time between 3 and 6 months, and 3 (3.6%) reported an overall treatment duration of approximately 1 year.

In the regenerative group, 29 of 45 respondents (64.4%) reported a treatment duration of approximately 1 year, 14 (31.1%) reported 3–6 months, and 2 (4.4%) reported functional loading within 72 h.

In the hybrid group, 18 of 27 respondents (66.7%) reported a treatment duration of 3–6 months, 6 (22.2%) reported approximately 1 year, and 3 (11.1%) reported functional loading within 72 h (Table 4).

**Table 4:** Estimated treatment duration according to preferred treatment strategy.

Treatment strategy	*n*	Within 72 h	3–6 months	∼1 year
Alternative implant strategies	83	69 (83.1%)	11 (13.3%)	3 (3.6%)
Regenerative approaches with bone grafting	45	2 (4.4%)	14 (31.1%)	29 (64.4%)
Hybrid/combined strategies	27	3 (11.1%)	18 (66.7%)	6 (22.2%)

Respondents selecting alternative implant strategies more frequently expected shorter treatment times and earlier functional loading, whereas respondents selecting regenerative approaches more commonly anticipated longer treatment duration. Hybrid approaches showed an intermediate pattern, most commonly associated with treatment times between 3 and 6 months.

### 3.7 Perceived aesthetic outcomes

Perceived aesthetic outcomes differed across therapeutic philosophies.

Regenerative approaches were more frequently associated with favorable aesthetic outcomes when volumetric reconstruction, labial support, and restoration of the soft-tissue profile were regarded as important treatment objectives.

However, a substantial proportion of respondents indicated that aesthetic outcomes may depend not only on the selected surgical technique, but also on prosthetic planning, implant positioning, soft-tissue management, and patient-specific anatomical characteristics.

Therefore, aesthetic outcomes should be interpreted as clinician-reported perceptions rather than objectively measured aesthetic results.

### 3.8 Aesthetic satisfaction with the smile

Clinician-reported satisfaction with the appearance of the smile was generally favorable across all therapeutic preference groups, although some differences emerged.

In the alternative implant strategies group, 69 of 83 respondents (83.1%) indicated that patients are satisfied with the appearance of their smile in most cases, while 14 (16.9%) considered satisfaction dependent on limited gingival exposure.

In the regenerative group, 39 of 45 respondents (86.7%) reported that patients are satisfied in most cases, whereas 6 respondents (13.3%) indicated that satisfaction may depend on the degree of gingival exposure.

In the hybrid group, 15 of 27 respondents (55.6%) reported satisfaction in most cases, 11 (40.7%) considered satisfaction dependent on limited gingival exposure, and 1 respondent (3.7%) indicated that additional aesthetic refinements are often required (Table 5).

**Table 5:** Smile aesthetics.

Treatment strategy	*n*	Satisfied	Dependent on gingival exposure	Additional refinements
Alternative implant strategies	83	69 (83.1%)	14 (16.9%)	0 (0.0%)
Regenerative approaches with bone grafting	45	39 (86.7%)	6 (13.3%)	0 (0.0%)
Hybrid/combined strategies	27	15 (55.6%)	11 (40.7%)	1 (3.7%)

Respondents selecting regenerative approaches most frequently reported favorable expectations regarding smile satisfaction with perceived aesthetic satisfaction, closely followed by alternative implant strategies. Hybrid approaches showed a more heterogeneous distribution of responses and were less consistently associated with favorable aesthetic outcomes.

### 3.9 Masticatory function

Recovery of masticatory function was generally perceived as favorable across all three therapeutic preference groups.

In the alternative implant strategies group, 53 of 83 respondents (63.9%) reported that chewing function was almost comparable to natural teeth with only minor limitations, while 28 (33.7%) considered it completely comparable to natural dentition. Only 2 respondents (2.4%) reported considerable functional differences.

In the regenerative group, 38 of 45 respondents (84.4%) indicated that chewing function was almost comparable to natural teeth with minor limitations, while 7 (15.6%) considered it completely comparable. No respondents reported considerable functional differences.

In the hybrid group, 14 of 27 respondents (51.9%) reported near-complete functional recovery, 10 (37.0%) considered chewing function completely comparable to natural dentition, and 3 respondents (11.1%) reported considerable functional differences (Table 6).

**Table 6:** Chewing function.

Treatment strategy	*n*	Almost comparable	Completely comparable	Considerable differences
Alternative implant strategies	83	53 (63.9%)	28 (33.7%)	2 (2.4%)
Regenerative approaches with bone grafting	45	38 (84.4%)	7 (15.6%)	0 (0.0%)
Hybrid/combined strategies	27	14 (51.9%)	10 (37.0%)	3 (11.1%)

Overall, the vast majority of respondents across all therapeutic preference groups reported complete or near-complete recovery of masticatory function. These findings indicate that respondents across all therapeutic preference groups generally expected satisfactory functional rehabilitation regardless of the selected therapeutic strategy. However, these data reflect clinician-reported perceptions and do not represent direct functional measurements or patient-reported outcomes.

### 3.10 Oral hygiene and maintainability

Oral hygiene and maintainability were generally considered manageable across all therapeutic preference groups, although some differences emerged.

In the alternative implant strategies group, 53 of 83 respondents (63.9%) reported that hygiene-related difficulties are mainly concentrated during the initial adaptation period and tend to stabilize over time. Twenty respondents (24.1%) considered hygiene management more demanding than with conventional implant-supported prostheses, while 10 (12.0%) reported no substantial differences.

In the regenerative group, 30 of 45 respondents (66.7%) indicated that hygiene-related challenges are primarily limited to the early postoperative phase, 10 (22.2%) considered maintenance more demanding, and 5 (11.1%) reported no relevant differences compared with conventional implant-supported rehabilitations.

In the hybrid group, 17 of 27 respondents (63.0%) reported that hygiene management becomes easier after an initial adaptation period, while 7 respondents (25.9%) considered maintenance more demanding and 3 (11.1%) reported no substantial differences (Table 7).

**Table 7:** Oral hygiene management.

Treatment strategy	*n*	Adaptation then stable	More demanding	No substantial differences
Alternative implant strategies	83	53 (63.9%)	20 (24.1%)	10 (12.0%)
Regenerative approaches with bone grafting	45	30 (66.7%)	10 (22.2%)	5 (11.1%)
Hybrid/combined strategies	27	17 (63.0%)	7 (25.9%)	3 (11.1%)

Overall, most respondents across all groups indicated that hygiene-related difficulties are mainly concentrated during the initial adaptation period and tend to stabilize over time. These findings suggest that oral hygiene and maintainability are perceived as important but generally manageable aspects of rehabilitation.

Overall, most respondents indicated that hygiene-related difficulties were mainly concentrated during the initial adaptation period and tended to stabilize over time.

### 3.11 Postoperative discomfort

Perceived postoperative discomfort differed among the three therapeutic preference groups.

In the alternative implant strategies group, 35 of 83 respondents (42.2%) reported an average duration of postoperative discomfort of less than one week, while 39 (47.0%) estimated discomfort lasting between one and four weeks. Only 9 respondents (10.8%) reported discomfort lasting longer than one month.

In the regenerative group, postoperative discomfort was generally perceived as more prolonged. Twenty-six of 45 respondents (57.8%) reported discomfort lasting between one and four weeks, while 12 (26.7%) estimated a duration exceeding one month. Only 7 respondents (15.6%) reported discomfort of less than one week.

In the hybrid group, 15 of 27 respondents (55.6%) reported discomfort lasting between one and four weeks, 7 (25.9%) reported discomfort lasting longer than one month, and 5 respondents (18.5%) reported symptoms resolving within one week (Table 8).

**Table 8:** Postoperative discomfort.

Treatment strategy	*n*	<1 week	1–4 weeks	>1 month
Alternative implant strategies	83	35 (42.2%)	39 (47.0%)	9 (10.8%)
Regenerative approaches with bone grafting	45	7 (15.6%)	26 (57.8%)	12 (26.7%)
Hybrid/combined strategies	27	5 (18.5%)	15 (55.6%)	7 (25.9%)

Overall, alternative implant strategies were more frequently associated with shorter postoperative discomfort, whereas regenerative and hybrid approaches were more commonly perceived as involving prolonged recovery periods. These findings reflect clinician-reported perceptions and should not be interpreted as direct measurements of postoperative morbidity.

### 3.12 Dropout due to morbidity

Across all therapeutic preference groups, treatment dropout attributable to surgical morbidity was most frequently estimated to be below 5%.

In the alternative implant strategies group, 67 of 83 respondents (80.7%) estimated dropout rates below 5%, while 14 (16.9%) estimated rates between 5% and 15%, and only 2 respondents (2.4%) estimated rates exceeding 15%.

In the regenerative group, 33 of 45 respondents (73.3%) estimated dropout rates below 5%, 11 (24.4%) estimated rates between 5% and 15%, and 1 respondent (2.2%) estimated rates above 15%.

In the hybrid group, 18 of 27 respondents (66.7%) estimated dropout rates below 5%, while 9 (33.3%) estimated rates between 5% and 15%. No respondents estimated dropout rates exceeding 15% (Table 9).

**Table 9:** Dropout.

Treatment strategy	*n*	<5%	5%–15%	>15%
Alternative implant strategies	83	67 (80.7%)	14 (16.9%)	2 (2.4%)
Regenerative approaches with bone grafting	45	33 (73.3%)	11 (24.4%)	1 (2.2%)
Hybrid/combined strategies	27	18 (66.7%)	9 (33.3%)	0 (0.0%)

These findings suggest that, although surgical morbidity is recognized as a relevant consideration during treatment planning, respondents generally estimated treatment discontinuation attributable to morbidity to be below 5%. As with the other survey domains, these data reflect clinician-reported perceptions rather than direct measurements of treatment adherence.

### 3.13 Annual maintenance costs

Annual maintenance costs were generally perceived as comparable across the three therapeutic preference groups, although some differences emerged.

In the alternative implant strategies group, 55 of 83 respondents (66.3%) considered annual maintenance costs comparable to those of other rehabilitation techniques, 22 (26.5%) considered them dependent on the specific patient or clinical situation, and 6 (7.2%) considered them higher because of the need for more frequent follow-up visits.

In the regenerative group, 36 of 45 respondents (80.0%) considered maintenance costs comparable, while 9 (20.0%) considered them dependent on the individual patient. No respondents in this group considered maintenance costs consistently higher.

In the hybrid group, 16 of 27 respondents (59.3%) considered maintenance costs comparable, 7 (25.9%) considered them patient-dependent, and 4 (14.8%) considered them higher because of the need for more frequent maintenance visits (Table 10).

**Table 10:** Maintenance costs.

Treatment strategy	*n*	Comparable	Patient-dependent	Higher
Alternative implant strategies	83	55 (66.3%)	22 (26.5%)	6 (7.2%)
Regenerative approaches with bone grafting	45	36 (80.0%)	9 (20.0%)	0 (0.0%)
Hybrid/combined strategies	27	16 (59.3%)	7 (25.9%)	4 (14.8%)

Overall, annual maintenance costs were most frequently perceived as comparable across therapeutic strategies. However, alternative implant and hybrid approaches were more frequently associated with patient-dependent variability and, in a minority of cases were more frequently associated with the expectation of higher maintenance requirements.

### 3.14 Clinician-reported patient satisfaction and quality of life

Clinician-reported patient satisfaction and perceived quality-of-life improvement were generally favorable across all therapeutic preference groups.

In the alternative implant strategies group, 66 of 83 respondents (79.5%) reported very high patient satisfaction (>90% of patients satisfied), while 16 (19.3%) reported moderate satisfaction levels. One respondent reported insufficient available data to estimate patient satisfaction. Similarly, 77 respondents (92.8%) reported a significant improvement in quality of life following treatment, 5 (6.0%) reported a moderate improvement, and 1 (1.2%) reported no noticeable change.

In the regenerative group, 37 of 45 respondents (82.2%) reported very high patient satisfaction, while 6 (13.3%) reported moderate satisfaction levels. Two respondents reported insufficient available data to estimate patient satisfaction. Forty-four respondents (97.8%) reported a significant improvement in quality of life, while 1 respondent (2.2%) reported a moderate improvement.

In the hybrid group, 21 of 27 respondents (77.8%) reported very high patient satisfaction, while 6 (22.2%) reported moderate satisfaction levels. Twenty-one respondents (77.8%) reported a significant improvement in quality of life, whereas 6 (22.2%) reported a moderate improvement (Tables 11, 12).

**Table 11:** Patient satisfaction.

Treatment strategy	*n*	Very high	Moderate	Insufficient data
Alternative implant strategies	83	66 (79.5%)	16 (19.3%)	1 (1.2%)
Regenerative approaches with bone grafting	45	37 (82.2%)	6 (13.3%)	2 (4.4%)
Hybrid/combined strategies	27	21 (77.8%)	6 (22.2%)	0 (0.0%)

**Table 12:** Quality of life.

Treatment strategy	*n*	Significant improvement	Moderate improvement	No improvement
Alternative implant strategies	83	77 (92.8%)	5 (6.0%)	1 (1.2%)
Regenerative approaches with bone grafting	45	44 (97.8%)	1 (2.2%)	0 (0.0%)
Hybrid/combined strategies	27	21 (77.8%)	6 (22.2%)	0 (0.0%)

Overall, respondents across all therapeutic preference groups reported high levels of patient satisfaction and substantial improvements in quality of life following rehabilitation. However, these outcomes were not assessed using direct patient-reported outcome measures or validated quality-of-life questionnaires and should therefore be interpreted as clinician-reported perceptions rather than direct patient-derived outcomes.

### 3.15 Summary of main findings

Overall, the survey demonstrated substantial variability in therapeutic preferences for the rehabilitation of severe maxillary atrophy among experienced clinicians, although alternative implant-based approaches represented the most frequently selected strategy.

Respondents selecting alternative implant strategies more frequently reported expectations of immediate loading, shorter treatment duration, higher predictability, and favorable implant survival. Respondents selecting regenerative approaches more frequently reported expectations related to volumetric reconstruction, soft-tissue support, and aesthetic rehabilitation, but were also perceived as requiring longer treatment times and delayed loading protocols.

Respondents selecting hybrid strategies generally described them as an intermediate and highly case-dependent therapeutic option.

Taken together, these findings provide insight into current international clinician-reported decision-making patterns in the management of severe maxillary atrophy. They should not be interpreted as evidence of clinical superiority of any specific technique, but rather as a reflection of contemporary expert preferences and perceptions.

## 4 Discussion

### 4.1 Principal findings

This international cross-sectional survey provides insight into current clinician-reported preferences and decision-making patterns in the rehabilitation of severe maxillary atrophy.

The most relevant finding of the present study is the absence of a single dominant therapeutic paradigm for the management of severe maxillary atrophy. Although alternative implant strategies represented the most frequently selected treatment option, regenerative and hybrid approaches also accounted for a substantial proportion of responses. This distribution suggests that participating clinicians perceive treatment planning for severe maxillary atrophy as increasingly individualized and influenced by multiple anatomical, prosthetic, functional, aesthetic, and patient-related factors.

The survey findings suggest that participating clinicians no longer view severe maxillary atrophy as requiring a single reconstructive philosophy. Instead, respondents reported selecting among alternative implant-based, regenerative, and hybrid strategies according to the perceived objectives of the standardized clinical scenario. In this context, the three therapeutic approaches should be regarded as complementary rather than mutually exclusive treatment options.

Alternative implant strategies were more frequently perceived as advantageous in terms of immediate loading, reduced treatment duration, predictability, and favorable expected implant survival (18). Regenerative approaches were more commonly associated with volumetric restoration, labial support, and aesthetic reconstruction (19), whereas hybrid approaches appeared to represent an intermediate and highly case-dependent solution integrating reconstructive and graftless principles (20).

Overall, the survey suggests that clinician-reported decision-making is primarily influenced by perceived treatment objectives and patient-specific considerations rather than adherence to a single preferred therapeutic philosophy.

### 4.2 Therapeutic preferences and contemporary treatment trends

The predominance of alternative implant strategies reported in the present survey may reflect the perceptions of experienced clinicians regarding the ongoing evolution of treatment concepts for severe maxillary atrophy. Participants selecting these approaches may have been influenced by advances in digital planning, guided surgery, remote anchorage concepts, and patient-specific implant design, which have expanded the range of graftless rehabilitation options. However, these findings should be interpreted as clinician-reported perceptions and therapeutic preferences derived from the standardized survey scenario rather than as evidence of current clinical practice or superiority of any specific treatment approach.

Alternative implant-based approaches, including zygomatic implants, pterygoid implants, transnasal implants, and patient-specific subperiosteal implants, allow rehabilitation by exploiting residual basal bone or remote skeletal anchorage sites (21, 22). Respondents selecting these approaches may associate them with reduced need for extensive bone reconstruction procedures and earlier functional rehabilitation. This perception is consistent with reports in the contemporary literature describing the increasing use of graftless rehabilitation strategies for severe maxillary atrophy.

The substantial proportion of respondents selecting regenerative or hybrid approaches suggests that many experienced clinicians continue to consider bone grafting an important component of treatment planning in selected clinical situations. Reconstruction of severely resorbed maxillae is often intended not only to create adequate implant support but also to restore skeletal volume, improve labial support, optimize prosthetic emergence profiles, and enhance soft-tissue architecture (23). These objectives may remain difficult to achieve through implant anchorage alone and continue to justify the use of reconstructive procedures in selected patients.

The results of the present survey therefore suggest that current clinical practice is characterized by coexistence rather than replacement of therapeutic philosophies. Alternative implant-based, regenerative, and hybrid approaches appear to address different clinical priorities and treatment objectives. Consequently, treatment selection is likely influenced by a complex interaction among anatomical conditions, prosthetic requirements, aesthetic expectations, treatment timing, morbidity considerations, and clinician experience.

Importantly, the present findings should not be interpreted as evidence that one therapeutic approach is superior to another. Rather, they provide insight into how experienced clinicians currently navigate the decision-making process when confronted with the same standardized clinical scenario.

### 4.3 Immediate loading, treatment duration, and predictability

One of the most evident differences among the therapeutic preference groups concerned the possibility of immediate loading and the overall duration of treatment.

Alternative implant strategies were more frequently associated with immediate loading and shorter treatment times. Respondents selecting alternative implant strategies frequently associated these approaches with immediate loading and shorter treatment times. This perception is consistent with the biological and prosthetic rationale described in the literature, in which implant anchorage is achieved through residual basal bone or remote skeletal structures, thereby reducing or eliminating the need for graft maturation and staged reconstruction procedures. As a result, treatment can often progress more rapidly from surgery to functional rehabilitation (24).

In contrast, regenerative approaches were more commonly associated with delayed loading protocols and longer overall treatment duration. This perception may reflect respondents' expectations regarding reconstructive treatment, which frequently involve bone augmentation, graft healing, implant placement, osseointegration, and subsequent prosthetic rehabilitation (25). Although these staged procedures may increase treatment time, they may also provide additional opportunities for volumetric reconstruction and prosthetically driven implant positioning.

Respondents selecting hybrid approaches most frequently associated these strategies with treatment durations between three and six months (26). This finding suggests that clinicians may adopt hybrid strategies when they seek to balance the advantages of graftless rehabilitation with specific reconstructive or prosthetic requirements.

Perceived predictability followed a similar pattern. Alternative implant strategies were more frequently considered predictable (27–29), whereas regenerative approaches were also commonly regarded as predictable but were associated with longer and more complex treatment pathways. These observations likely reflect differences in clinician experience, familiarity with specific techniques, and expectations regarding treatment complexity rather than direct comparisons of clinical performance.

Taken together, these findings suggest that treatment timing remains a major determinant of therapeutic decision-making in severe maxillary atrophy. The possibility of reducing the number of surgical stages and accelerating functional rehabilitation appears to represent an important factor influencing clinician preferences. However, shorter treatment duration should not be interpreted as an indicator of overall treatment superiority, as different therapeutic approaches may pursue distinct reconstructive and prosthetic objectives.

### 4.4 Aesthetic considerations

Aesthetic rehabilitation remains one of the most challenging aspects of treatment planning in patients with severe maxillary atrophy. In these cases, the clinical objective often extends beyond implant placement and functional restoration, encompassing reconstruction of the alveolar process, support of the upper lip, restoration of facial contours, and optimization of peri-implant soft-tissue architecture (30).

In the present survey respondents selecting regenerative approaches more frequently perceived these techniques as advantageous for aesthetic rehabilitation. This observation is likely related to the ability of reconstructive procedures to restore hard- and soft-tissue volume, thereby facilitating prosthetically driven implant placement and improving support of the peri-oral soft tissues.

At the same time, according to respondents that aesthetic outcomes are not exclusively dependent on the selected surgical technique. Prosthetic design, implant positioning, emergence profile management, soft-tissue conditioning, and patient-specific anatomical characteristics were all considered important determinants of the final aesthetic result. These perceptions reinforce the view among participating clinicians that the concept that aesthetic success should be regarded as a multidisciplinary objective requiring integration of surgical and prosthetic planning rather than the automatic consequence of a particular therapeutic approach.

Interestingly, smile satisfaction was reported as generally favorable across all therapeutic preference groups. This suggests that different treatment philosophies may achieve satisfactory aesthetic outcomes when appropriately applied to the specific anatomical and prosthetic requirements of individual patients. Consequently, the present findings support a treatment-planning model in which aesthetic objectives are balanced with functional requirements, treatment duration, morbidity considerations, and patient expectations.

Because aesthetic outcomes were not directly measured and no objective aesthetic indices were collected, these observations should be interpreted as clinician-reported perceptions rather than comparative evidence of aesthetic superiority among treatment modalities.

### 4.5 Patient satisfaction, function, and quality of life

Clinician-reported patient satisfaction, perceived masticatory function, and quality-of-life improvement were generally favorable across all therapeutic preference groups. Although differences emerged in treatment philosophy, duration, and rehabilitation protocols, respondents consistently expected rehabilitation of severe maxillary atrophy to provide substantial functional and psychosocial benefits.

Masticatory function was reported as complete or nearly complete in the vast majority of responses across all groups. This finding indicates that participating clinicians generally expected that alternative implant-based, regenerative, and hybrid approaches may all provide effective restoration of oral function when treatment planning and execution are appropriately adapted to the clinical scenario. Importantly, respondents generally considered restoration of chewing ability as an achievable objective regardless of the selected therapeutic pathway.

Similarly, patient satisfaction was reported as high across all groups, with most clinicians indicating that patients are satisfied with both functional and aesthetic outcomes following rehabilitation.

Quality-of-life improvement was also consistently perceived as substantial. This finding is particularly relevant because rehabilitation of severe maxillary atrophy often affects domains extending beyond oral function alone, including speech, facial appearance, social interaction, self-confidence, and psychological well-being (31–33).

However, these findings must be interpreted with caution. Neither patient satisfaction nor quality of life were assessed directly using validated patient-reported outcome measures (PROMs). Instead, the present results reflect clinician perceptions derived from professional experience and routine clinical practice. Consequently, they should not be considered equivalent to direct patient-reported outcomes.

Future research should incorporate validated instruments assessing oral-health-related quality of life, patient satisfaction, mastication, speech, aesthetics, and psychosocial well-being in order to better understand the patient perspective and to complement clinician-reported perceptions.

### 4.6 Clinical implications

The present survey may have several implications for understanding clinician decision-making in severe maxillary atrophy.

First, the survey findings suggest that many experienced clinicians support an individualized approach to treatment planning. The wide distribution of preferences observed among experienced clinicians suggests that multiple rehabilitation strategies may be considered appropriate depending on the specific anatomical, functional, prosthetic, and aesthetic requirements of each patient (34, 35).

Second, the survey indicates that participating clinicians consider individualized decision-making. The choice between alternative implant-based, regenerative, and hybrid approaches appears to be influenced not only by the severity of bone loss but also by factors such as treatment duration, possibility of immediate loading, expected morbidity, soft-tissue requirements, prosthetic objectives, patient expectations, and clinician experience.

Third, the findings emphasize the importance of multidisciplinary planning. Respondents emphasized the importance of integrating surgical, prosthetic, functional, and aesthetic considerations during treatment planning.

Overall, the survey suggests that participating clinicians favor an individualized, objective-driven treatment-planning philosophy rather than adherence to a single preferred technique.

### 4.7 Strengths, limitations, and future perspectives

The present study has several strengths. To the authors' knowledge, it represents one of the largest international surveys specifically focused on clinician decision-making in the rehabilitation of severe maxillary atrophy. The inclusion of experienced clinicians from 33 countries provided a broad overview of contemporary therapeutic preferences and contributed to the international relevance of the findings.

Another strength is the use of a standardized anonymized CBCT case, which allowed all participants to evaluate the same clinical scenario. This approach reduced variability related to case selection and facilitated direct comparison of therapeutic preferences among respondents with different professional backgrounds and geographic origins.

Nevertheless, several limitations should be acknowledged. First, the study was designed as a cross-sectional survey and therefore reflects clinician-reported opinions, preferences, and perceptions rather than objective clinical outcomes. Consequently, the findings should not be interpreted as evidence of superiority, effectiveness, or long-term performance of any specific rehabilitation strategy.

Second, participation was voluntary and recruitment was performed through professional, academic, and scientific networks. As a result, selection bias and self-selection bias cannot be excluded.

Third, all outcome domains evaluated in the survey were based on clinician perceptions rather than prospective patient data, validated patient-reported outcome measures, or direct clinical follow-up.

An additional limitation relates to the branching structure of the questionnaire. Because respondents evaluated only the therapeutic approach they selected as their preferred strategy, comparisons among groups reflect differences in clinician perceptions rather than direct comparative assessments of multiple treatment modalities by the same respondents.

Future research should complement clinician-reported surveys with prospective clinical studies, multicenter registries, and investigations incorporating validated patient-reported outcome measures.

Accordingly, the findings should be interpreted as reflecting clinician-reported perceptions, expectations, and therapeutic preferences elicited by a standardized clinical scenario rather than direct evidence of contemporary clinical practice or comparative effectiveness of the evaluated rehabilitation strategies.

## 5 Conclusions

This international cross-sectional survey provides insight into contemporary clinician-reported preferences and decision-making patterns in the rehabilitation of severe maxillary atrophy.

The findings demonstrate substantial variability in therapeutic preferences among experienced clinicians, with alternative implant-based approaches representing the most frequently selected strategy, while regenerative and hybrid approaches continue to play an important role in treatment planning.

Overall, the results suggest that contemporary management of severe maxillary atrophy is increasingly based on individualized treatment planning rather than adherence to a single therapeutic philosophy.

Because the present study reflects clinician-reported perceptions rather than direct clinical outcomes, the findings should not be interpreted as evidence of superiority of any specific rehabilitation strategy. Future prospective studies incorporating objective clinical outcomes and validated patient-reported outcome measures are needed to further clarify the relationship between clinician preferences and real-world treatment results.

## Data Availability

The original contributions presented in the study are included in the article/Supplementary Material, further inquiries can be directed to the corresponding author.

## The Collaborative Working Group

Department of Periodontics and Preventive Dentistry, School of Dental Medicine, University of Pittsburgh, Pittsburgh, Pennsylvania, USA: Martinna Bertolini. Department of Periodontics and Preventive Dentistry, School of Dental Medicine, University of Pittsburgh, Pittsburgh, Pennsylvania, USA: Cristiano Soares Moreira. Department of Oral Medicine, Infection, & Immunity, Harvard School of Dental Medicine: Shayan Barootchi. Private Practice, Fresno CA: Ryan Andrew Dunlop. Department of Orofacial Sciences, School of Dentistry, University of California, San Francisco, USA: Guo-Hao Lin. Department of Oral and Maxillofacial Surgery, University of Arizona PHX Campus, Banner University Medical Center, Phoenix, Arizona, USA: Vishtasb Broumand. Departamento de Cirugia Oral y Maxilofacial Universidad de la Republica Oriental del Uruguay, Hospital Italiano Montevideo Uruguay: Felipe Kornecki Radzinski. Department of Periodontology, School of Dentistry, University of Başkent, Ankara, Turkey: Emine Elif Alaaddinoglu. Department of Oral and Maxillofacial Surgery, Faculty of Dentistry, Recep Tayyip Erdogan University, Rize, Türkiye: Alperen Kalyouncu. Department of Periodontology, Faculty of Dentistry, Gazi University, Ankara, Türkiye: Sila Cagri Isler. Second Dental Center, Shanghai Ninth People's Hospital, Shanghai Jiao Tong University, Shanghai, China: Yiqun Wu. Smart Arches Dental Implant Centers USA: Simon Oh. Department of Oral and Maxillofacial Surgery, Faculty of Dentistry, University of Dicle, Diyarbakır, Turkey: Ridvan Guler. Department of Oral and Maxillofacial Surgery, Faculty of Dentistry, İzmir Katip Çelebi University, İzmir, Türkiye: Onur Şahin. Department of Periodontology, School of Dentistry, University of Başkent, Ankara, Turkey: Engin Ozgur. INNOTERE Biomaterials, Radebeul, Germany: Sascha Heinemann. Department of Oral Health & Medicine, University Center for Dental Medicine Basel (UZB), University of Basel, Basel, Switzerland: Michael Marc Bornstein. Department of Oral Surgery and Stomatology, University of Bern School of Dental Medicine, Bern, Switzerland: Emilio Couso-Queiruga. Department of Oral Surgery and Stomatology, University of Bern School of Dental Medicine, Bern, Switzerland: Clemens Raabe. Perio-Implant Innovation Center, Institute of Integrated Oral, Craniofacial and Sensory Research, Shanghai Ninth's People Hospital, Shanghai Jiao Tong School of Medicine, Shanghai, China: Andrea Roccuzzo. Clinic of Reconstructive Dentistry, Center for Dental Medicine, University of Zurich, Zurich, Switzerland: Daniel Thoma. State Key Laboratory of Oral Diseases, National Center for Stomatology, National Clinical Research Center for Oral Diseases, West China Hospital of Stomatology, Sichuan University, Chengdu, China: Yi Man. Department of Oral Biochemistry, Institute of Odontology, The Sahlgrenska Academy at University of Gothenburg, Gothenburg, Sweden; Farah Asa'ad. Department of Odontology, Umeå University, Sweden: Mats Sjöström. Department of Periodontology, College of Dentistry, Yonsei University, Seoul, South Korea: Jae-Kook Cha. Private Practice, Centre for Implantology and Oral Medicine, Johannesburg, South Africa: Muhammed Hanif Abram. Head of Clinical Department of Maxillofacial Surgery, Russian University of Medicine, Moscow, Russia: Nicolai Redko. Head of Clinical Department of Maxillofacial Surgery, Russian University of Medicine, Moscow, Russia: Alexey Drobyshev. Head of Clinical Department of Maxillofacial Surgery, Russian University of Medicine, Moscow, Russia: Eduard Kharazyan. Private Practice, Florence, Italy: Cesare Paoleschi. Wickham Dental Care, Carseldine QLD, Australia: Sadesh Kumar. Department of Propaedeutics of Dental Diseases, Faculty of Dentistry, Bashkir State Medical University, Ufa, Russia: Andrey Trokhalin. Department of Maxillofacial and Plastic Surgery, BV Petrovsky Russian Scientific Center of Surgery, Moscow, Russia: Ilya Bozo. Department of Oral and Maxillofacial Surgery, Fogolyán Kristóf Emergency County Hospital, Sfantu Gheorghe, Romania: Zoltán Fábián. Department of Implantology, Apollonia University Iasi, Iasi, Romania: Dragan Eliza. Victor Babeș University of Medicine and Pharmacy Timișoara, Timișoara, Romania: Mariu Leretter. Department of Oral and Maxillofacial Surgery, Clitrofa-Centro Médico, Dentário e Cirúrgico, Trofa, Portugal: Fernando Manuel Pinto Duarte. Diamante Dental Clinics, Lubin/Zielona Góra, Poland: Adam Aleksnder Nowicki. University of Oslo, Oslo, Norway: Ilaria Vitali. Department of Oral and Maxillofacial Surgery, Treant Hospital Scheper, Emmen, The Netherlands: Jurjen Schortinghuis. Department of Oral and Maxillofacial Surgery, Erasmus MC University Medical Center, Rotterdam, the Netherlands: Justin Pijpe. Department of Oral and Maxillofacial Surgery, University of Groningen, University Medical Center Groningen, Groningen, The Netherlands: Gerry M Raghoebar. Department of Oral and Maxillofacial surgery, Micunovic medical clinic, Podgorica, Montenegro: Miljan Micunovic. Department of Odontology and periodontology “Sofia Sirbu”,USMF “Nicolae Testemitanu”, Chișinău, Moldavia: Viorica Chetrus. Private practice, Meliva VIC klinikos, Vilnius, Lithuania: Algirdas Puisys. Assistant Lecturer of Prosthetic Dentistry, Alasmarya Islamic University, Zliten, Libya: Mohammad Ibrahim Alghraisi. Department of Maxillofacial Surgery, Policlinico di Bari, Bari, Italy: Stefan Cocis. Department of Oral Surgery, School of Dentistry, University of Padova: Rocco Borrello. Department of Oral Surgery, School of Dentistry, University of Padova: Alessandro Paccagnella. Department of Maxillofacial Surgery, University of Siena, Siena, Italy: Lisa Catarzi. Department of Maxillofacial Surgery, University of Siena, Siena, Italy; Division of Maxillofacial Surgery, Marche University Hospitals- Umberto I, Ancona, Italy: Giulio Cirignaco. Department of Dentistry, Catholic University of the Sacred Heart, University of Rome, Rome, Italy: Alessandro Ciolfi. Department of Oral and Maxillofacial Surgery, Aalborg University Hospital, Aalborg, Denmark: Thomas Starch-Jensen. Department of Oral and Maxillofacial Surgery, Aalborg University Hospital, Aalborg, Denmark: Hanna Cecilia Aludden. Department of Oral Surgery, Gabriele d’Annunzio University of Chieti-Pescara, Italy: Paolo Ricci. Department of Oral Surgery, Gabriele d'Annunzio University of Chieti-Pescara, Italy: Stefania Romano. Department of Maxillofacial Surgery Spedali Civili, Brescia, Italy: Stefano Negrini. Integrated Service Maxillofacial Surgery/Head-Neck Surgery, Nefrocenter Healthcare System and Hospital Group, Adjunct Assistant Professor Dept. ENT/Head-Neck Surgery University of Maryland School of Medicine - Baltimore, USA: Vito del Deo. Division of Maxillofacial Surgery, Marche University Hospitals- Umberto I, Ancona, Italy: Giuseppe Consorti. Department of Oral and Maxillofacial Surgery, County Hospital “San Bortolo”, Vicenza, Italy: Riccardo Girotto. Private Practice, Genova, Italy: Roberto Rossi. Private practice, Gasport, NY, USA: Igor Kaplasky. Department of Head and Neck and Neurosciences, Maxillofacial Surgery Unit, San Camillo-Forlanini Hospital, Rome, Italy: Flavio Andrea Govoni. Department of Head and Neck and Neurosciences, Maxillofacial Surgery Unit, San Camillo-Forlanini Hospital, Rome, Italy: Roberto Pistilli. Department of General Sugery and Surgical-Medical Specialties, School of Dentistry, University of Catania, 95123 Catania, Italy: Gaetano Isola. Division of Maxillofacial Surgery, Marche University Hospitals- Umberto I, Ancona, Italy: Andrea Balercia. Department of Oral and Maxillofacial Sciences, Sapienza University of Rome, Italy: Edoardo Brauner. Department of Neuroscience, School of Dentistry, University of Padova, Padova, Italy: Stefano Sivolella. Unit of Oral and Maxillofacial Surgery and Odontostomatology, University of Verona, Verona, Italy: Gianluca Colapinto. Department of Oral and Maxillofacial Surgery, School of Dentistry, Internation University of Catalonia, Barcelona, Spain: Adaia Valls-Ontañón. Department of Oral surgery and implantology, Faculty of Medicine and Health Sciences, University of Barcelona, Barcelona, Spain: Adria Jorba-Garcia. Department of Oral surgery and implantology, Faculty of Medicine and Health Sciences, University of Barcelona, Barcelona, Spain: Octavi Camps-Font. Nano lab, Eye Transitional Research center, farabi Eye Hospital, Tehran University of Medical sciences, Tehran Iran: Ahad Khoshzaban. Department of Oral and Maxillofacial Surgery, “Diaz” Private Hospital, Padova, Italy: Aldo Zupi. Department of Oral and Maxillofacial Surgery, University Hospital LKH Salzburg, Salzburg, Austria: Alexander Johann Gaggl. Oral Surgery, Nantes University and University Hospital, Nantes, France: Anne-Gaëlle Chaux. Department of Oral and Maxillofacial Surgery, Federal Armed Forces Hospital, Koblenz, Germany; Oral and Maxilloafacial Surgery, University Medical Center; J Gutenberg University, Mainz Germany: Anne-Kathrin Bär. Oral and Maxilloafacial Surgery, University Medical Center; J Gutenberg University, Mainz Germany: Bilal Al-Nawas. Unit of Maxillofacial Surgery, Regional Hospital F Miulli, University of Bari, Acquaviva delle Fonti, Bari, Italy: Antonio Mancini. Department of Oral Surgery, Gabriele d'Annunzio University of Chieti-Pescara, Italy: Antonio Scarano. Department of Oral and Maxillofacial Surgery, Ziekenhuis aan de stroom (ZAS), Antwerp, België: Casper Van den Borre. Private practice, Rome, Italy: Christian Alberti. Department of Oral and Maxillofacial Surgery and Centre for Reconstructive Surgery, University Hospital of the Private Medical University Paracelsus LKH, Salzburg, Austria: Christoph Steiner. Department of Oral and Maxillofacial Surgery and Centre for Reconstructive Surgery, University Hospital of the Private Medical University Paracelsus LKH, Salzburg, Austria: Gian Battista Bottini. Department of Oral and Maxillofacial Surgery, Head and Neck Universitary Institute, Nice Hospital, Nice, France: Cyril Debortoli. Integral Dentistry Department, Faculty of Dentistry, Universidad de La Frontera, Temuco, Chile: Eduardo Borie. Department of Dental Clinical Specialties. School of Dentistry. University Complutense of Madrid: Eduardo Montero. Department of Dentistry, Faculty of Medical Sciences, Albanian University, Tirana, Albania: Erda Qorri. Department of Oral and Maxillofacial and Periodontology, Ribeirão Preto School of Dentistry, University of São Paulo, Ribeirão Preto, Brazil: Erick Ricardo Silva. Department of Oral and Maxillofacial and Periodontology, Ribeirão Preto School of Dentistry, University of São Paulo, Ribeirão Preto, Brazil: Giuseppe Alexandre Romito. Faculty of Dental Sciences, Institute of Medical sciences.Banaras Hindu University.Varanasi, India: Farhan Durrani. Department of Oral and Maxillofacial Surgery, Royal Melbourne Hospital and Geelong University of Hospital: Felix Sim. Division of Oral Surgery, University Clinic of Dentistry, Medical University of Vienna, Vienna, Austria: Florian Beck. Department of Surgical Sciences, University of Turin, Turin, Italy: Giacomo Baima. Private practice Bad Oeynhausen, Germany: Jochen Tunkel. Department of Oral and Maxillofacial Surgery - Plastic Operations, University of Schleswig-Holstein, Campus Kiel: Henning Wieker. Department of Oral and Maxillofacial Surgery, Helios Hospitals, Kassel, Germany: Hendrik Terheyeden. Periodontology, Centre for Dental Education and Research, All India Institute of Medical Sciences, New Delhi, India: Kunaal Dhingra. Department of digital oral implantology and prosthodontics, College of Stomatology, Xi'an Jiaotong University, Xi'an, 710004, China: Liangzhi Du. Maxillo-Facial Surgery Unit, Department of Medicine, Surgery and Pharmacy, University of Sassari, Sassari, Italy: Luigi Angelo Vaira. Department for Oral and Maxillofacial Surgery, School of Dentistry, University of Dresen (TUD), Germany: Manfred Nilius. Dentistry Department, School of Health and Life Sciences, Pontifical Catholic University of Rio Grande do Sul (PUCRS), Porto Alegre, Brazil: Marcel Ferreira Kunrath. Department of Prosthodontics, Faculty of Dentistry, Cairo University, Giza, Egypt: Mariam Ahmed Roshdy. Department of Operative and Esthetic Dentistry, Faculty of Dentistry, University of Szeged, Szeged, Hungary: Mark Adam Antal. Private practice's owner and freelencer: Gregorio Redaelli. Department of of prosthodontics, faculty of dentistry, Mansoura University Mansoura, EGYPT: Mohamed Elgamal. Oral and Maxillofacial Department, Faculty of Dentistry, Menoufia University: Mohamed T Khater. Department of Prosthodontics, Faculty of Dentistry, Menoufia National University, Menoufia, Egypt: Mohammed A El-Sawy. Department of Oral Medicine, Periodontology and Oral Diagnosis, Faculty of Dentistry, AinShams University, Cairo, Egypt: Mohammed Samir. Department of Implant Dentistry, College of Stomatology, Xi'an Jiaotong University: Ningbo Zhao. CL Dentistry, Private Practice, Segni, Italy: Paolo Carosi. Department of Prosthodontics, New Horizon Dental College & Research Institute, Bilaspur, Chattisgarh, India: Gaurav Tripathi. Department of Periodontics and Oral Medicine, University of Michigan School of Dentistry, Ann Arbor, MI, USA: Hamoun Sabri. Department of Periodontics and Oral Medicine, University of Michigan School of Dentistry, Ann Arbor, MI, USA: Hom-Lay Wang.
