# Supplementary material for: Clinician preferences and decision-making patterns in the rehabilitation of severe maxillary atrophy: an international cross-sectional survey using a standardized cawood and howell class VI case
Source: Front Oral Health. 2026 Aug 4;7:1903798. doi: 10.3389/froh.2026.1903798 (PMC13481304; doi:10.3389/froh.2026.1903798)
Supplement: Supplementary file 1 [file Supplementaryfile1.docx]

**Supplementary Material 1**

**Complete Survey Questionnaire**

Title of the survey: Maxillary Atrophy Class VI Cawood and Howell: How to Perform Implant-Prosthetic Rehabilitation?

This supplementary material reports the complete questionnaire used for the international cross-sectional survey. The questionnaire was administered through Google Forms and included an eligibility screening question, author/contact information fields, a standardized clinical case, general decision-making questions, and branching sections according to the therapeutic strategy selected by the respondent.

**Section 1. Eligibility Screening**

**1. How many complex rehabilitations did you complete in 2024?**

Response format: Single-choice

- <5
- 5–10
- >10

*Note: Respondents reporting fewer than five complex rehabilitations per year were directed to an exit section and excluded from the final analysis.*

**Section 2. Participation and Author Information**

**2. Do you agree to participate in this collaborative study?**

Response format: Single-choice

- Yes
- No

**3. Full name**

Response format: Free text

*Note: Format requested: first name, middle name/initial if applicable, last name.*

**4. Institutional affiliation**

Response format: Free text

*Note: Department/Unit, Faculty/Division, University/Hospital/Institution, City, Country.*

**5. Email address**

Response format: Free text

*Note: Professional/institutional email preferred; personal email accepted.*

**6. ORCID iD**

Response format: Free text

*Note: Optional but recommended.*

**Section 3. Standardized Clinical Case**

Respondents were presented with a standardized case of an 82-year-old female patient with severe maxillary atrophy requiring fixed implant-supported rehabilitation. The clinical information provided included sex, age, ASA status, relevant medical history, current medications, financial considerations, and the patient request for fixed implant-supported prosthetic rehabilitation.

A link to the anonymized CT/CBCT dataset was provided in the online questionnaire to allow respondents to evaluate the case using their own planning systems.

**7. Confirmation of access to the CT/CBCT scan**

Response format: Checkbox

- Ok

**Section 4. General Therapeutic Preference Questions**

**8. In this case, what is your preferred technique?**

Response format: Single-choice

- Bone grafting (autograft/xenograft) + traditional implants
- Alternative implantology (pterygoid/zygomatic/transnasal/tilted/subperiosteal/piezoimplant)
- Combination of both
- Other

**9. What factors most influence your choice?**

Response format: Multiple-choice

- Rehabilitation time
- 5-year implant survival rate
- Costs for the patient
- Surgical complexity
- Other

**10. In your experience, which technique provides the best aesthetic results?**

Response format: Single-choice

- Bone graft + traditional implants
- Alternative implantology (e.g., zygomatic/pterygoid)
- No difference
- I don't have sufficient data

**11. Which approach has, according to you, the highest 5-year survival rate in severe atrophies?**

Response format: Single-choice

- Bone grafts + traditional implants
- Alternative implants
- I don't have sufficient data

**12. Which technique, according to you, requires more long-term prosthetic revisions?**

Response format: Single-choice

- Bone grafts + traditional implants
- Alternative implants (e.g., All-on-4/zygomatic)
- No difference
- I don't have sufficient data

**13. The occlusal load distribution is more physiological with:**

Response format: Single-choice

- Traditional implants in axial position
- Alternative implants with proper balancing
- I don't notice clinical differences

**14. Choose your preferred rehabilitation technique in the provided case.**

Response format: Single-choice

- Alternative implant
- Bone graft and traditional implant
- Both
- Bone graft, traditional implant and alternative implant where necessary

*Note: This item determined the branching pathway for the subsequent strategy-specific questions.*

**Branch A. Hybrid/Combined Strategy**

**1. Could you please provide a brief description of your implant-prosthetic project?**

Response format: Free text

**2. Is your technique:**

Response format: Multiple-choice

- Safer (lower intraoperative risks)
- More predictable (better control of the outcome)
- Well tolerated by the patient (high morbidity)
- Not applicable in these cases
- Other

**3. Does your technique allow for immediate loading with a fixed prosthesis in complex cases?**

Response format: Single-choice

- Yes, always/usually
- Only in selected cases
- No, it requires a long time

**4. What is the expected 5-year survival rate for your technique in severe atrophy?**

Response format: Single-choice

- >95%
- 85–94%
- <85%

**5. Does your technique require complex/expensive prosthetics?**

Response format: Single-choice

- Yes, always
- No, standard
- Depends on the case

**6. Is your technique more prone to prosthetic complications (e.g., fractures, screw loosening)?**

Response format: Single-choice

- Yes, frequently
- No, it is stable
- Only in case of planning errors

**7. Considering the use of the same materials for each technique, is your chosen technique more expensive compared to other techniques?**

Response format: Single-choice

- Yes, significantly
- No, it costs the same as others
- Depends on the materials used

**8. The total treatment time, from surgery to functional loading, is:**

Response format: Single-choice

- <72 hours (immediate loading)
- 3–6 months
- 1 year

**9. In your experience, what is the patient satisfaction level 1 year after prosthetic loading?**

Response format: Single-choice

- Very high (>90% of patients satisfied)
- Moderate (70–90% satisfied)
- Low (<70% satisfied)
- Data not available

**10. The majority of post-treatment complaints involve:**

Response format: Multiple-choice

- Pain/prosthetic discomfort
- Aesthetic problems (e.g., unnatural gingival profile)
- Chewing/speech difficulties
- Surgical complications (e.g., infections)
- Other

**11. How much does the quality of life (Oral Health Impact) improve for patients after treatment?**

Response format: Single-choice

- Significant improvement
- Moderate improvement
- No noticeable change

**12. Do patients report difficulty with daily oral hygiene with your technique?**

Response format: Single-choice

- Yes, often (requires special tools)
- Only initially
- No, it is as simple as with natural teeth

**13. Are patients satisfied with the appearance of their smile (naturalness, lip profile)?**

Response format: Single-choice

- Yes, in most cases
- Only if the upper lip does not expose the gum
- No, they often require touch-ups

**14. Is the chewing function comparable to natural teeth?**

Response format: Single-choice

- Yes, completely
- Almost, with minor limitations
- No, considerable differences

**15. The post-operative discomfort lasts on average:**

Response format: Single-choice

- <1 week
- 1–4 weeks
- 1 month

**16. How many patients abandon treatment due to high morbidity?**

Response format: Single-choice

- <5%
- 5–15%
- >15%

**17. Is the annual maintenance more expensive compared to other techniques?**

Response format: Single-choice

- Yes, for frequent check-ups
- No, it is comparable
- Depends on the patient

**Branch B. Alternative Implant Strategies**

**1. Could you please provide a brief description of your implant-prosthetic project?**

Response format: Free text

**2. Is your technique:**

Response format: Multiple-choice

- Safer (lower intraoperative risks)
- More predictable (better control of the outcome)
- Well tolerated by the patient (high morbidity)
- Not applicable in these cases
- Other

**3. Does your technique allow for immediate loading with a fixed prosthesis in complex cases?**

Response format: Single-choice

- Yes, always/usually
- Only in selected cases
- No, it requires a long time

**4. What is the expected 5-year survival rate for your technique in severe atrophy?**

Response format: Single-choice

- >95%
- 85–94%
- <85%

**5. Does your technique require complex/expensive prosthetics?**

Response format: Single-choice

- Yes, always
- No, standard
- Depends on the case

**6. Is your technique more prone to prosthetic complications (e.g., fractures, screw loosening)?**

Response format: Single-choice

- Yes, frequently
- No, it is stable
- Only in case of planning errors

**7. Considering the use of the same materials for each technique, is your chosen technique more expensive compared to other techniques?**

Response format: Single-choice

- Yes, significantly
- No, it costs the same as others
- Depends on the materials used

**8. The total treatment time, from surgery to functional loading, is:**

Response format: Single-choice

- <72 hours (immediate loading)
- 3–6 months
- 1 year

**9. In your experience, what is the patient satisfaction level 1 year after prosthetic loading?**

Response format: Single-choice

- Very high (>90% of patients satisfied)
- Moderate (70–90% satisfied)
- Low (<70% satisfied)
- Data not available

**10. The majority of post-treatment complaints involve:**

Response format: Multiple-choice

- Pain/prosthetic discomfort
- Aesthetic problems (e.g., unnatural gingival profile)
- Chewing/speech difficulties
- Surgical complications (e.g., infections)
- Other

**11. How much does the quality of life (Oral Health Impact) improve for patients after treatment?**

Response format: Single-choice

- Significant improvement
- Moderate improvement
- No noticeable change

**12. Do patients report difficulty with daily oral hygiene with your technique?**

Response format: Single-choice

- Yes, often (requires special tools)
- Only initially
- No, it is as simple as with natural teeth

**13. Are patients satisfied with the appearance of their smile (naturalness, lip profile)?**

Response format: Single-choice

- Yes, in most cases
- Only if the upper lip does not expose the gum
- No, they often require touch-ups

**14. Is the chewing function comparable to natural teeth?**

Response format: Single-choice

- Yes, completely
- Almost, with minor limitations
- No, considerable differences

**15. The post-operative discomfort lasts on average:**

Response format: Single-choice

- <1 week
- 1–4 weeks
- 1 month

**16. How many patients abandon treatment due to high morbidity?**

Response format: Single-choice

- <5%
- 5–15%
- >15%

**17. Is the annual maintenance more expensive compared to other techniques?**

Response format: Single-choice

- Yes, for frequent check-ups
- No, it is comparable
- Depends on the patient

**Branch C. Bone Grafting and Traditional Implants**

**1. Could you please provide a brief description of your implant-prosthetic project?**

Response format: Free text

**2. Is your technique:**

Response format: Multiple-choice

- Safer (lower intraoperative risks)
- More predictable (better control of the outcome)
- Well tolerated by the patient (high morbidity)
- Not applicable in these cases
- Other

**3. Does your technique allow for immediate loading with a fixed prosthesis in complex cases?**

Response format: Single-choice

- Yes, always/usually
- Only in selected cases
- No, it requires a long time

**4. What is the expected 5-year survival rate for your technique in severe atrophy?**

Response format: Single-choice

- >95%
- 85–94%
- <85%

**5. Does your technique require post-operative surgical touch-ups?**

Response format: Single-choice

- Yes, frequently
- Rarely
- Almost never

**6. Is your technique more prone to prosthetic complications (e.g., fractures, screw loosening)?**

Response format: Single-choice

- Yes, frequently
- No, it is stable
- Only in case of planning errors

**7. Considering the use of the same materials for each technique, is your chosen technique more expensive compared to other techniques?**

Response format: Single-choice

- Yes, significantly
- No, it costs the same as others
- Depends on the materials used

**8. The total treatment time, from surgery to functional loading, is:**

Response format: Single-choice

- <72 hours (immediate loading)
- 3–6 months
- 1 year

**9. In your experience, what is the patient satisfaction level 1 year after prosthetic loading?**

Response format: Single-choice

- Very high (>90% of patients satisfied)
- Moderate (70–90% satisfied)
- Low (<70% satisfied)
- Data not available

**10. The majority of post-treatment complaints involve:**

Response format: Multiple-choice

- Pain/prosthetic discomfort
- Aesthetic problems (e.g., unnatural gingival profile)
- Chewing/speech difficulties
- Surgical complications (e.g., infections)
- Other

**11. How much does the quality of life (Oral Health Impact) improve for patients after treatment?**

Response format: Single-choice

- Significant improvement
- Moderate improvement
- No noticeable change

**12. Do patients report difficulty with daily oral hygiene with your technique?**

Response format: Single-choice

- Yes, often (requires special tools)
- Only initially
- No, it is as simple as with natural teeth

**13. Are patients satisfied with the appearance of their smile (naturalness, lip profile)?**

Response format: Single-choice

- Yes, in most cases
- Only if the upper lip does not expose the gum
- No, they often require touch-ups

**14. Is the chewing function comparable to natural teeth?**

Response format: Single-choice

- Yes, completely
- Almost, with minor limitations
- No, considerable differences

**15. The post-operative discomfort lasts on average:**

Response format: Single-choice

- <1 week
- 1–4 weeks
- 1 month

**16. How many patients abandon treatment due to high morbidity?**

Response format: Single-choice

- <5%
- 5–15%
- >15%

**17. Is the annual maintenance more expensive compared to other techniques?**

Response format: Single-choice

- Yes, for frequent check-ups
- No, it is comparable
- Depends on the patient

**Exit Section**

Respondents who completed the questionnaire or were not eligible according to the predefined experience threshold were directed to a final thank-you message.
